# Sgo1 is a potential therapeutic target for hepatocellular carcinoma

**DOI:** 10.18632/oncotarget.2764

**Published:** 2015-01-06

**Authors:** Lyu-Han Wang, Chia-Jui Yen, Tian-Neng Li, Sabine Elowe, Wen-Ching Wang, Lily Hui-Ching Wang

**Affiliations:** ^1^ Institute of Molecular and Cellular Biology, National Tsing Hua University, Hsinchu, Taiwan; ^2^ Université Laval, Faculty of Medicine, Department of Pediatrics, and Reproduction, Perinatal Health, and Infant Health, CHUL-CRCHUQ, 2705 Blvd. Laurier, T-3-67, Québec G1V 4G2, Canada; ^3^ Institute of Clinical Medicine, National Cheng Kung University, Tainan, Taiwan; ^4^ Department of Medical Science, National Tsing Hua University, Hsinchu, Taiwan

**Keywords:** Shugoshin, Hepatocellular Carcinoma, Hepatitis B Virus, Large Surface Antigen, Mitosis, Catastrophe, Cohesin

## Abstract

Shugoshin-like protein 1 (Sgo1) is an essential protein in mitosis; it protects sister chromatid cohesion and thereby ensures the fidelity of chromosome separation. We found that the expression of Sgo1 mRNA was relatively low in normal tissues, but was upregulated in 82% of hepatocellular carcinoma (HCC), and correlated with elevated alpha-fetoprotein and early disease onset of HCC. The depletion of Sgo1 reduced cell viability of hepatoma cell lines including HuH7, HepG2, Hep3B, and HepaRG. Using time-lapse microscopy, we showed that hepatoma cells were delayed and ultimately die in mitosis in the absence of Sgo1. In contrast, cell viability and mitotic progression of immortalized cells were not significantly affected. Notably, mitotic cell death induced upon Sgo1 depletion was suppressed upon inhibitions of cyclin-dependent kinase-1 and Aurora kinase-B, or the depletion of mitotic arrest deficient-2. Thus, mitotic cell death induced upon Sgo1 depletion in hepatoma cells is mediated by persistent activation of the spindle assembly checkpoint. Together, these results highlight the essential role of Sgo1 in the maintenance of a proper mitotic progression in hepatoma cells and suggest that Sgo1 is a promising oncotarget for HCC.

## INTRODUCTION

During mitosis, fidelity of chromosome segregation depends on precise control of sister chromatids separation in space and time. The maintenance of sister chromatid cohesion is one essential mechanism to ensure that premature chromosome separation does not occur prior to the onset of anaphase [1, 2]. Shugoshin-like protein 1 (Sgo1) is known as the “guardian spirit', and protects centromeric cohesion from premature dissolution by mitotic kinases and Wapl [3–7]. HeLa cells depleted of Sgo1 displayed premature chromosome separation and therefore committed cell death as a result of prolonged arrest in a prometaphase-like stage [8, 9]. Although Sgo1 is clearly an essential protein in mitosis, its role in carcinogenesis remains controversial. Overexpression of Sgo1 has been reported in breast and pancreatic cancers [10, 11]. On the other hand, Sgo1 expression is decreased in colorectal cancer, and the depletion of Sgo1 caused G2/M arrest, apoptosis, and chromosome instability in a colon cancer cell line [12, 13]. While splicing variants of Sgo1 have been implicated centrosome instability and chromosome instability in colon and lung cancers [14–16], the role of the full-length Sgo1 in cancers has not been investigated.

Hepatocellular carcinoma (HCC) develops with complex genetic backgrounds, and severe chromosome allelic losses and structural aberrations simultaneously contribute to genomic heterogeneity in advanced stages of HCC [17–19]. The heterogeneity of HCC not only makes it difficult for prognosis but also for the development of effective systemic therapeutic agents for advanced HCC [20]. Thus, it is urgent to understand the mechanisms underlying the severe genomic and chromosome instability that triggers the development of HCC and to search for new therapeutic targets and pathways for new drug developments.

In the present study, we investigated the expression level of Sgo1 in various normal tissues and HCC specimens. In addition, we explored the essential roles of Sgo1 in the maintenance of cell survival on transformed HeLa cells and four different hepatoma cell lines, as well as two immortalized cell lines. Our results indicated that, in comparison to immortalized cells, transformed hepatoma cells were more sensitized to spindle assembly checkpoint (SAC)-dependent mitotic cell death induced upon Sgo1 depletion. These results highlight the essential role of Sgo1 in supporting proper mitotic progression and cell viability in hepatoma cells and therefore suggested that Sgo1 is a valuable therapeutic target for HCC.

## RESULTS

### Sgo1 is upregulated in transformed hepatoma cells

**Figure 1:**
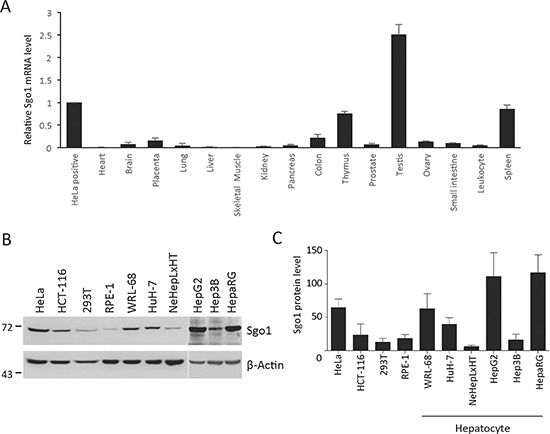
Tissue and cell line expression profiles of Sgo1 **(A)** Quantitative real-time PCR analysis of Sgo1 mRNA in HeLa cells and 16 normal tissues as indicated. The Y-axis represents the fold change in the expression of Sgo1 mRNA relative to that in HeLa cells and after normalization with levels of gapdh. **(B)** Protein expressions of Sgo1 and β-actin were examined in eight transformed cell lines (HeLa, HCT-116, 293T, WRL-68, and HuH-7, HepG2, Hep3B, ad HepaRG) and two immortalized cell lines (RPE-1 and NeHepLxHT). **(C)** Quantitation results of Sgo1 protein levels in these cell lines were shown. Asterisks represent a significance difference by Student's t-test (*, *P* < 0.05; **, *P* < 0.01).

To investigate the status of Sgo1 expression in various human tissues, we measured the mRNA level of Sgo1 by quantitative real-time-PCR using primers covering the full-length A1/A2 isoforms. Sgo1 mRNA was mostly expressed in thymus, testis, and spleen. The mRNA level of Sgo1 is low in the majority of normal tissues, including heart, brain, placenta, lung, liver, skeletal muscle, kidney, pancreas, colon, prostate, ovary, small intestine, and leukocytes (Fig. 1A). Next, to explore the potential role of Sgo1 in the development of human malignancies, we compared Sgo1 protein expression in eight transformed (HeLa, HCT-116, 293T, WRL-68, HuH-7, HepG2, Hep3B, and HepaRG) and two immortalized (RPE-1 and NeHepLxHT) cell lines expressing human telomerase reverse transcriptase. Sgo1 was detected in all cell lines with different expression levels (Fig. 1B). Notably, among the six hepatocyte-derived cell lines, hepatoma cell lines expressed 3-10-fold higher levels of Sgo1 when compared with immortalized NeHepLxHT cells (Fig. 1C). Similarly, RPE-1 cells displayed a relatively lower level of Sgo1 when compared other transformed cells. Together, all eight transformed cell lines displayed a relatively higher level of Sgo1 in comparison to two non-transformed cell lines.

### Upregulation of Sgo1 in hepatocellular carcinoma

**Figure 2:**
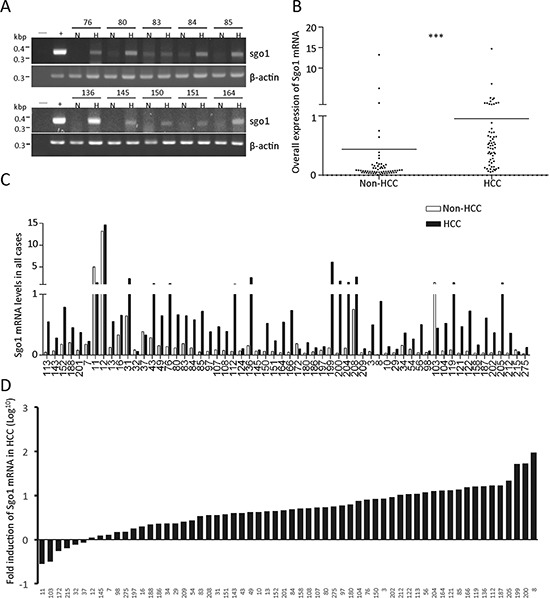
Messenger RNA expression profile of Sgo1 in HCC and adjacent non-HCC tissues **(A)** Representative electrophoresis results of conventional Sgo1 RT-PCR obtained from ten paired HCC (H) and adjacent non-HCC (H) specimens. Numbers shown above indicate case identification numbers. **(B)** Quantitative expressions levels of Sgo1 mRNA in HCC and non-HCC specimens are plotted. All expression levels are normalized to that of *gapdh* in each sample and are expressed as the fold-increase for each sample relative to HeLa cells, which was set at 1. Asterisks represent a significance difference by Student's t-test (***, *p* < 0.001). **(C)** Relative expression levels of Sgo1 mRNA in all cases were plotted. **(D)** Expression level of Sgo1 mRNA in HCC was plotted as fold change (in Log^10^) relative to that in adjacent non-HCC tissues.

To explore the clinicopathological significance of Sgo1, we examined Sgo1 mRNA in 60 HCC and adjacent non-HCC liver tissues by both conventional RT-PCR and quantitative real-time PCR. By conventional PCR, Sgo1 and actin mRNA was detected in 97% (58/60) of HCC and 73% (44/60) of adjacent livers (Fig. 2A). Next, relative copy numbers of Sgo1 mRNA in HCC and non-HCC tissues was measured by quantitative real-time PCR, using HeLa cDNA as a batch standard and gapdh as the internal control (Fig. 2B). Although the expression levels of Sgo1 varied among different cases (Fig. 2C), Sgo1 mRNA was upregulated (>1.5-fold) in 82% (49/60) cases of HCC (Fig. 2D). We compared various clinical parameters in HCC patients with different levels of Sgo1 expression. Sixty patients were divided into Sgo1-Low and Sgo1-High groups according to the ranking of Sgo1 mRNA levels in HCC. Statistically, HCC patients in the Sgo1-High group were significantly younger than patients in the Sgo1-Low group. In addition, serum alpha-fetoprotein (AFP) level was also significantly higher in the Sgo1-High group (Table 1). Besides these two parameters, the level of Sgo1 in HCC did not correlate with sex, liver cirrhosis, hepatitis B virus (HBV) positivity, vascular invasion, tumor size, differentiation and staging. In addition, we did not find a significant difference in the disease-free survival after surgery between both groups. Together, these results indicated that upregulation of Sgo1 is associated with an early disease onset of HCC.

**Table 1:** Clinical parameters of HCC with low and high levels of Sgo1 mRNA

	Whole group (*n* = 60)	Sgo1-Low^a^ (*n* = 30)	Sgo1-High^a^ (*n* = 30)	*P*
Age (year)^b^	58.83; 57 (33–82)	62.33; 61.5 (37–82)	55.33; 56.5 (33–76)	0.0119^c^
Sex (male)	44 (73%)	24 (80%)	20 (67%)	0.3817
AFP (ng/ml)	2496; 13.12 (2–60500)	120; 8.46 (2–1768)	4873; 42.86 (2-60500)	0.0321^c^
HBV positivity	40 (66.7%)	17 (57%)	23 (77%)	0.0851
Vascular invasion	27 (45%)	11 (37%)	16 (33%)	0.1497
Liver cirrhosis	9 (15%)	6 (20%)	3 (10%)	0.2358
Tumor diameter (cm)	5.87; 6 (1–17)	6.17; 6 (3–8)	5.59; 4.4 (1–17)	0.2144
Tumor differentiation				0.4891
Well	6 (10%)	2 (7%)	4 (13%)	
Moderate	44 (73%)	24 (80%)	20 (67%)	
Poor	10 (17%)	4 (13%)	6 (20%)	
Tumor staging				0.6058
I	24 (40%)	14 (50%)	11 (37%)	
II	23 (38%)	10 (33%)	13 (43%)	
III	11 (18%)	6 (17%)	5 (17%)	
IV	1 (2%)	0	1 (3%)	

**Abbreviations:** AFP, alpha-fetoprotein; HBV, hepatitis B virus; Sgo1-Low, Sgo1 expression level below 50% of tumor regions; Sgo1-High, Sgo1 expression higher than 50% of tumor regions

a Sixty cases were separated into two groups according to the Sgo1 mRNA level in HCC. Sgo1-Low and Sgo1-High groups represent Sgo1 mRNA level in the lower and top 50% of total HCC cases, respectively.

b Age, AFP, tumor volume, tumor size is expressed as mean, medium, and range. All other parameters are expressed as count with percentage shown in brackets.

c Age and serum AFP levels have statistically significant associations with a high Sgo1 level in HCC.

**Figure 3:**
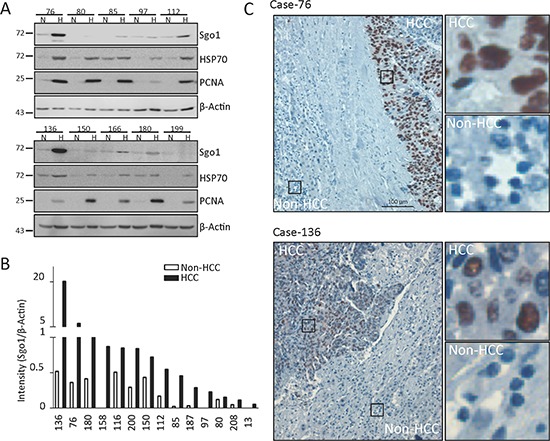
Sgo1 protein expression in HCC and adjacent non-HCC tissues **(A)** Representative blots of Sgo1 and β-actin in ten paired HCC (H) and adjacent non-HCC (N) specimens. **(B)** Quantitative expressions levels of Sgo1 protein in HCC and non-HCC specimens are plotted after normalization with actin levels. Sgo1 expression level was increased in 14 HCCs in comparison to adjacent non-HCC regions. **(C)** Representative immunohistochemistry images illustrating that Sgo1 expressed in cell nuclei of HCC but not in adjacent liver tissues. Right panels showed enlarged images from boxed areas as indicated.

We further examined protein expressions of Sgo1in lysates prepared from 21 paired-HCC and adjacent non-HCC tissues, using heat shock protein 70 (HSP70) and proliferating cell nuclear antigen (PCNA) as positive controls for HCC (Fig. 3A). Whereas HSP70 was detected in 14 (66%) cases of HCC, PCNA was detected in 20 (95%) cases of HCC. Here, Sgo1 protein was detected in 71% (15/21) HCC and 57% (12/21) non-HCC tissues (Table 2). Among these Sgo1-positive cases, 87% (13/15) of HCC tissues displayed relatively higher level (>1.5 fold) of Sgo1 in comparison to adjacent normal tissues (Fig. 3B). It should be noted that protein lysates prepared from HCC tissues might still contain adjacent non-HCC regions. Therefore, the upregulation level of Sgo1 in HCC is likely underestimated when evaluated by conventional western blots. To gain more insight into the expression of Sgo1 in HCCs, immunohistochemical staining was performed on 10 paraffin-embedded specimens containing HCC and adjacent non-HCC tissues. Sgo1 was detected in the cell nuclei in all 10 HCC tissues (Fig. 3C). In adjacent non-HCC tissues, Sgo1 was only weakly detected in three cases and completely undetectable in other seven cases. Together, these results supported the upregulation of Sgo1 protein expression in hepatocellular carcinoma.

**Table 2:** Comparison of Sgo1 protein expression levels^a^ in HCC and adjacent non-HCC

	HCC (*n* = 21)	Non-HCC (*n* = 21)	*P*
Sgo1 positivity^b^	15 (71%)	12 (57%)	0.2602
Average Sgo1 expression^c^	358.9 ± 180.5	32.62 ± 11.39	0.0394^d^

a Sixty micrograms of lysates extracted from HCC or non-HCC specimens were resolved by SDS-PAGE, the Sgo1 signal was visualized by Western blotting, and signal intensity was measured by chemiluminescence detection and quantitated by Image Quant.

b Number and the percentage of cases with a detective level of Sgo1 protein in western blots are shown.

c Average signal intensity of Sgo1 on the blots after normalization to actin.

d Sgo1 expression level (relative pixel intensity after normalization with actin levels) in HCC is significantly higher than that in non-HCC tissues.

### Sgo1 deficiency induced mitotic cell death in transformed hepatoma cells

**Figure 4:**
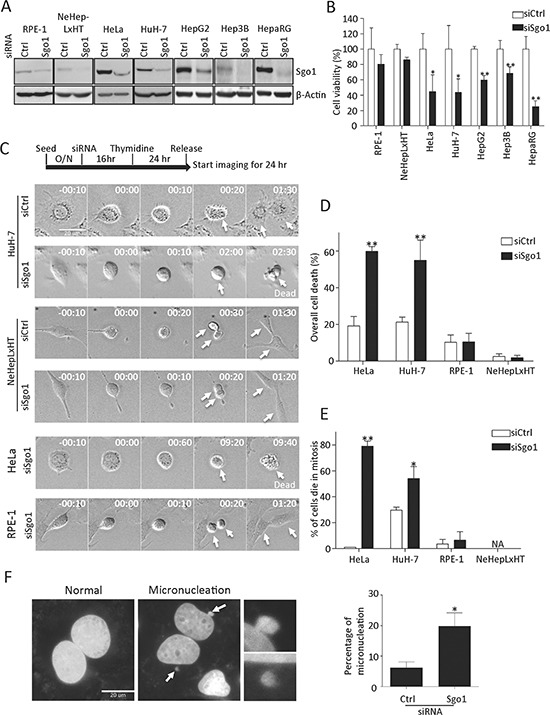
Sgo1 depletion reduced cell viabilities in HeLa and various hepatoma cells **(A)** Endogenous levels of Sgo1 in two immortalized and five different transformed cell lines were reduced upon the depletion Sgo1 by RNA interference. Blot for β-actin was included as an internal control. **(B)** Cell viabilities of indicated cell lines treated with control or Sgo1 siRNA were examined by WST-1 assay. **(C)** Mitotic progressions of HuH-7, NeHepLxHT, HeLa, and RPE-1 cells depleted with Sgo1 were monitored by time-lapse live cell microscopy. Cells were synchronized with thymidine and released after 24 hours when acquisition of differential interference contrast images began as illustrated by the experimental design. Representative time-point images were selected to show mitotic entry (time stamps displayed as 00:00 for hr:min) and progression of indicated cell lines. White arrows followed the same cell and its progenies over time within same panels. Overall number of cell death **(D)** and cells death in mitosis **(E)** during time-lapse imaging. **(F)** HuH-7 cells were treated with control and Sgo1 siRNA for 48 hrs followed by fixation and staining with 4′,6-diamidino-2-phenylindole (DAPI). Representative images of cells without or with micronucleation (indicated by white arrows, enlarged images shown in right panels). The depletion of Sgo1 increased micronucleation by approximately 3-fold in interphase cells. Asterisks represent a significance difference by Student's t-test (*, *p* < 0.05; **, *p* < 0.01).

Sgo1 is essential for sister chromatid cohesion during mitosis and its depletion has been shown to induce cell death in cervical carcinoma and lung cancer cells [8, 21]. With regard to the relatively high expression level of Sgo1 observed in HCC and hepatoma cell lines, we suggest that hepatoma cells may demand a high level of Sgo1 for the maintenance of sister chromatid cohesion. To test this notion, we investigated cell proliferation and mitotic cell progression in the absence of Sgo1. Using a defined small interfering RNA oligonucleotide targeting Sgo1 [7], we successfully reduced endogenous levels of Sgo1 by 60~85% in two immortalized cell lines (RPE-1 and NeHepLxHT) and five different transformed cell lines (HeLa, HuH-7, HepG2, Hep3B, HepaRG) (Fig. 4A). Cell viability of these five transformed cells was significantly reduced upon Sgo1 depletion. In contrast, Sgo1 depletion had no significant impact on RPE-1 and NeHepLxHT cells (Fig. 4B). To understand the nature of reduced cell viability in these transformed cells, we followed cell cycle progression by time-lapse microscopy. Upon Sgo1 depletion, both HuH-7 and HeLa cells displayed a delay in mitosis for several hours and ultimately, these cells died without exiting mitosis, a phenomenon known as mitotic catastrophe (Fig. 4C, 4D). Specifically, about 80% of HeLa cells and 50% of HuH-7 cells were delayed in mitosis for hours before ultimately dying (Fig. 4E). We noted that micronucleation, another morphological trait of mitotic catastrophe, was significantly increased in HuH-7 cells upon Sgo1 depletion (Fig. 4F). In striking contrast, immortalized NeHepLxHT and RPE-1 cells progressed through mitosis without a significant defect upon Sgo1 depletion (Fig. 4C, 4D). Similar results were obtained in other three hepatoma lines (data not shown). Together, these results highlight the crucial role of Sgo1 in the maintenance of cell viability and a proper mitotic progression in transformed hepatoma cells.

### Sgo1 depletion caused checkpoint-dependent cell death and the loss of sister chromatid cohesions in hepatoma cells

**Figure 5:**
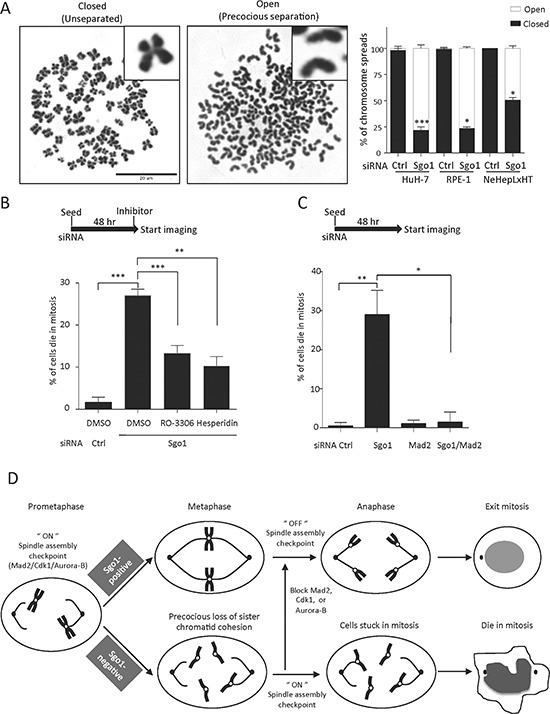
Sgo1 depletion induced precocious separation and SAC-dependent mitotic cell death **(A)** HuH-7, RPE-1 and NeHepLxHT cells were treated with control and Sgo1 siRNA for 48 hours and mitotic cells collected for chromosome spreading and stained with DAPI. Representative images for cells carrying closed (non-separated) or open (precocious separated) chromatids were shown as indicated. Percentage of cells displayed closed or open chromosome configuration is shown on the right panel. **(B)** Mitotic cell death in HuH-7 cells induced by Sgo1 depletion was reduced upon treatments with 5 μg/ml RO-3306 or 100 nM Hesperidin. **(C)** Mitotic cell death in HuH-7 cells upon Sgo1 depletion was rescued by the co-depletion of Mad2. *, *p* < 0.05; **, *p* < 0.01; ***, *p* < 0.001. **(D)** In normal cells, the SAC monitors both tension across sister centromeres and attachments between kinetochores and microtubules. Only after proper chromosome biorientation is achieved in all sister chromatids, the spindle assembly checkpoint is turned off to allow anaphase onset. In the absence of Sgo1, sister chromatid cohesion is lost prematurely before checkpoint silencing. As a result, cells are stuck in mitosis for hours and ultimately die without exiting mitosis. By applying inhibitors of Cdk1 and Aurora-B, or the co-depletion with Mad2, the SAC is silenced to allow anaphase onset in cell depleted of Sgo1. Thus, mitotic cell death in the absence of Sgo1 depends on the integrity of SAC.

In HeLa cells, the depletion of Sgo1 resulted in precocious dissociation of cohesin complex from centromeres and thereby prevented the silencing of the SAC. As a result, cells were unable to exit mitosis and arrested in a prometaphase-like state for several hours [7, 8]. To explore whether Sgo1 is also involved in sister chromatid cohesion in hepatoma cells, we examined wwprecocious sister chromatid separation upon the depletion of Sgo1 in HuH-7 cells (Fig. 5A). Similarly, premature loss of sister chromatid cohesion was detected in immortalized RPE-1 and to a lesser extent in NeHepLxHT cells (Fig. 5A). Thus, Sgo1 is essential for sister chromatid cohesion in both transformed and immortalized cells. We next investigated whether SAC mediates mitotic cell death induced upon Sgo1 depletion. To this end, we treated asynchronized HuH-7 cells with Sgo1 siRNA for 48 hours. At this stage, about 50% of the cell populations were stuck in mitosis, as judged by the typically rounded mitotic cell shape. Time-lapse imaging was used to record the fate of these mitotic cells after 48 hours of siRNA transfection. Within a 10-hour time window, about 27% of mitotic cells died in mitosis in the absence of Sgo1 (Fig. 5B), whereas in control cells, only about 1.7% of mitotic cells died during the imaging period. Treatments with RO-3306 or Hesperidin to block cyclin-dependent kinase-1 (Cdk1) and Aurora kinase-B (Aurora-B), respectively, at the beginning of time-lapse imaging significantly reduced the number of cells dying in mitosis (Fig. 5B). This suggested that mitotic cell death was mediated by the activation of SAC. To further support this notion, mitotic arrest deficient-2 (Mad2), a key regulator of the SAC, was depleted in HuH-7 cells. Mitotic cell death induced by Sgo1 depletion was rescued by the co-depletion of mitotic arrest deficient-2 (Mad2), (Fig. 5C). These results demonstrate essential roles of Sgo1 in the maintenance of sister chromatid cohesion and thereby contribute to the silencing of the SAC. Together, these results indicate that Sgo1 may serve a novel mitotic target for HCC.

## DISCUSSION

The present study identified Sgo1 as a promising and novel therapeutic target for HCC. Sgo1 mRNA was barely detected in the majority of normal tissues but was significantly expressed in various transformed hepatoma cell lines. Notably, upregulation of Sgo1 mRNA was detected in 82% of HCC tissues in comparison to adjacent non-HCC regions. In addition, nuclear staining of Sgo1 was detected in all HCC specimens, whereas adjacent livers displayed either weak or no Sgo1 staining. In addition, higher level of Sgo1 mRNA in HCC were linked to younger age and elevated serum alpha-fetoprotein, suggesting that upregulation of Sgo1 may contribute to an early disease onset of HCC. The most intriguing finding in the present study is the distinct sensitivity to Sgo1 depletion among hepatoma cell lines and immortalized cells. This suggests that hepatoma cells generally require a high level of Sgo1 to support proper mitotic progression and cell proliferation. Accordingly, Sgo1 may serve a selective therapeutic target for HCC without causing a significant defect on adjacent non-transformed hepatocytes.

Besides HCC, as shown in this study, upregulation of Sgo1 was found in expression datasets of breast and pancreatic cancers [10, 11, 22]. The expression level of Sgo1 was also elevated in leukemia cell lines derived from hematological malignancies [23]. On the other hand, downregulation of Sgo1 has been linked to chromosome instability in colorectal cancer [12, 13]. In addition, mice with Sgo1 haploinsufficiency display dysregulated centrosome dynamics and an increased chromosome instability, whereas the Sgo1 knockout is embryonic lethal [12]. Thus, it is plausible that Sgo1 plays distinct roles in the development different human malignancies. This should be taken into consideration in the development of future targeting therapeutic approaches.

Several splice variants of the Sgo1 gene have been predicted but whether these mRNA variants are constantly spliced and translated in the cell remains unclear [8, 16, 24]. A small C2 isoform, also known as sSgo1, was shown to function in centriole cohesion [14]. In colon cancer, a tumor-derived P1 variant was identified as a negative regulator of the full-length Sgo1 in the cell [16]. Recently, overexpression of Sgo1 variant B was found in non-small cell lung cancer. Ectopic expression of the SgoL1-B induced abnormal mitosis and resistance to taxol in non-small cell lung cancer cell line [15]. It should be noted that endogenous protein expressions of these small variants were never verified in above-mentioned studies. We have investigated the presence of Sgo1 splicing variants in different cell lines and normal tissues (Supplementary information). Although these splicing mRNA variants were detected, whether these small variants are translated into functional proteins remains unclear. At the protein level, a major product of about 70 kDa corresponding to the full-length A1/A2 isoforms of Sgo1 was consistently detected in our (this study) and other laboratories using either monoclonal or polyclonal antibodies raised again the conserved region of Sgo1 [8, 9, 25]. Our attempts to detect these smaller isoforms in HeLa and hepatoma cells were not successful (Supplementary information). However, this does not exclude the possibility that small isoforms are indeed expressed but in a much lower level and have therefore escaped detection.

Our findings indicate that Sgo1 is a common survival factor for hepatoma cells. During mitosis, Sgo1 is involved in the protection of centromeric cohesion by counteracting the actions of mitotic kinases and Wapl, through its association with protein phosphatase 2A [3–7, 26]. In eukaryotes, the SAC prevents chromosome mis-segregation by monitoring stable tension across centromeres and the attachment across sister kinetochores and microtubules [27]. Checkpoint signaling in prometaphase results in the formation of the mitotic checkpoint complex (MCC), which negatively regulates the anaphase-promoting complex/cyclosome and thereby blocks the mitotic progression into anaphase. The generation of MCC involves Mad genes, such as Mad1, Mad2, and BubRI, and several kinases including Cdk1, Aurora-B, and multipolar spindle-1 (Mps1) [28]. As shown in this study, the depletion of Sgo1 resulted in premature loss of sister chromatid cohesion (Fig. 5). This was sensed by the MCC and therefore cells were stuck in a prometaphase-like stage for hours until committed cell death (Fig. 4C). In agreement with this, a small peptide contain a C-terminus fragment of Sgo1 was proposed to act as a dominant inhibitor of Sgo1 and induced mitotic arrest in HeLa and A549 cells [21]. We show that the inactivation of the SAC, by inhibitions of Cdk1 and Aurora-B or the depletion of Mad2, could suppress mitotic cell death induced by Sgo1 depletion (Fig. 5B and 5C). These results indicated that mitotic catastrophe induced by the depletion of Sgo1 is mediated by the activation of the SAC. Therefore, the checkpoint mechanism that evolved to ensure the fidelity of chromosome segregation can now be utilized to selectively target transformed cells with chromosome instabilities.

It is not clear why hepatoma cells are more sensitive to Sgo1 depletion than immortalized cells. We suspect that chromosome instability in these cells may serve a genuine explanation. During mitosis, timely dissolution of sister chromatid cohesion ensures accurate chromosome segregation to guard against chromosome instability and tumorigenesis. Recent study suggests that Sgo1 binds to centromeric cohesin in a complex containing protein phosphatase 2A (PP2A), PDS5, and hypophosphorylated soronin, and thereby protects cohesin from the WAPL-mediated dissolution [29]. It is plausible that cells with extra chromosomes may need more Sgo1 to preserve sister chromatid cohesion. Over 90% of RPE-1 and NeHepLxHT contain a modal chromosome number of 46 [30, 31]. In contrast, HeLa cells contain one extra version of most chromosomes, with up to five copies of some, and an average chromosome number of 82 [32]. An average of 55 and 90 chromosomes was detected in HuH-7 and HepG2 cells in our laboratory (data not shown) and others [33]. The correlation between the need for Sgo1 and the number of chromosomes remains to be clarified in further studies. Notably, sister chromatid cohesion was also lost in RPE-1 and NeHepLxHT cells upon Sgo1 depletion. As these immortalized cells were able to pass through mitosis in the absence of Sgo1, this indicates that immortalized cells are more resistant to situations that activate spindle assembly checkpoint. Practically, the distinct response to mitotic checkpoint activation among normal and cancer cells have provided the window to selectively target cancer cells by various anti-mitotic agents [34, 35].

The idea of targeting cancer cells through the activation of spindle assembly checkpoint is being actively exploited. Vinca alkaloids block microtubule polymerization and thereby prevent the formation of a proper spindle during mitosis, which subsequently lead to mitotic catastrophe [34]. Inhibitors of different mitotic regulators, such as cyclin-dependent kinases, Aurora kinase, and Polo-like kinase, have been developed and evaluated *in vitro* and in animal models, and some of them have reached clinical trials [36]. So far, these novel kinase inhibitors have shown limited efficacy in clinical trials and classical anti-microtubule drugs are still the best approach in targeting mitosis to fight cancer [36]. Thus, the identification of new therapeutic target is required for a more efficient and safer way to fight cancer cells in mitosis. The present study suggests that Sgo1 is a promising therapeutic target for HCC.

## METHODS

### Cell lines and tissue samples

HeLa, HuH-7, WRL-68, HepG2, Hep3B, 293T, HCT-116, and RPE-1 cells were grown in DMEM (Biowest) with 10% fetal bovine serum and 1% penicillin/streptomycin. NeHepLxHT cells were purchased from ATCC (American Type Culture Collection, USA) and maintained under the suggested conditions (see details in supplementary information). HepaRG cells were purchased from Invitrogen (Life Technologies, Carlsbad, CA, USA) and maintained as suggested by the supplier. All cells were incubated at 37°C in a humidified atmosphere with 5% CO_2_. Human multiple tissue panels of cDNA were obtained from Clontech (catalog nos. 636742 and 636743). Paired HCC and adjacent non-HCC liver tissues were obtained from National Cheng Kung Hospital (Tainan, Taiwan) with prior approval from the Institutional Review Board (approval number: B-ER-102-154).

### RNA extraction, RT-PCR, and quantitative real-time PCR

Total RNA was extracted with Trizol (Invitrogen) and reverse-transcribed to cDNA using SuperScript III (Invitrogen). The resulting cDNA was applied for conventional reverse-transcription (RT)-polymerase chain reaction (PCR) using the following primers: Sgo1, ACCAGGCCTCTAGCTAAAAGAGCAC and CGTCTCAAATCCTTTTTCTGCTTGA; actin, ATCATGTTTGAGACCTTCAA and CATCTCTTGCTCGAAGTCCA. For quantitation, cDNA prepared from cell lines or clinical tissues were amplified by the following primers: Sgo1, AACCTGCTCAGAACCAGGAA and CTGGAGCTGTCATCACTATTGG; gapdh, CAAGGCTGTGGGCAAGGT and GGAAGGCCATGCCAGTGA. Real-time PCR was used Power SYBR^®^ Green PCR Master Mix (ABI) in ABI StepOnePlus™ Real-Time PCR Systems. The cycling steps are 95°C 10 min, 40 cycles of 95°C 15 s and 60°C 1 min.

### Immunohistochemistry staining

Paraffin-embedded samples were pre-warmed at 65°C for 15 min, followed by treatment with xylene to remove the paraffin. The samples were rehydrated by sequential incubation in 100%, 95%, and 70% ethanol and then in water. Antigen retrieval was performed by boiling the samples in tissue pretreatment buffer solution (Invitrogen) for 30 min. The slides were then incubated with mouse anti-Sgo1 antibody (Abnova) for 1 hour and detected using the EnVision+ System-HRP kit (DAKO). The samples were counterstained with hematoxylin (DAKO) and imaged under a Zeiss LSM780 laser confocal microscope or a Leica DMI6000 microscope.

### Transfection of siRNA and western blotting

RNA transfections were performed using Oligofectamine or RNAiMAX (Invitrogen) according to the manufacturer's instructions. A control GL2 siRNA (siCtrl) and a siRNA targeting Sgo1 were ordered from Dharmacon (Standard) using published sequences [7, 37]. To analyze the cellular proteins by western blotting, the cells were lysed in RIPA buffer (50 mM Tris-HCl [pH 7.5], 150 mM NaCl, 0.1% SDS, 1% NP-40, 0.5% sodium deoxycholate, and protease inhibitors) and resolved by sodium dodecyl sulfate polyacrylamide gel electrophoresis, transferred to a PVDF membrane, and incubated with mouse anti-Sgo1 (Abnova) or actin (Norvus), followed by detection using the enhanced chemiluminescence method. The resulting data were analyzed and quantified using ImageQuant software (GE Healthcare).

### Cell viability assay

We measured the cell proliferation activity with the water-soluble tetrazolium salt (WST-1) assay (Clontech). Cells were seeded in 96-well microtiter plates at a concentration of 2,000 cells/well. The WST-1 reagent was added to the cultures for 2.5 hr and the absorbance of samples was measured at 450 nm against the background using 650 nm as the reference wavelength.

### Time-lapse live cell microscopy

Cells were cultured in 3.5-cm dishes (BD) or 4-well chamber slides (ibidi). Multi-positional time-lapse imaging was performed using a Leica DMI6000 inverted microscope equipped with an HCX PL FL 20x/NA0.4 objective and an Andor Luca R EMCCD camera. DIC images were used to examine cell morphology and mitotic progression with a 10-min interval time for 24 hours. Image analysis and cell counts were performed using Metamorph software (Molecular Devices).

### Chromosome spreads

Cells were mitotic shaken-off and then treated with a hypotonic solution of 70 mM KCL for 15-min at room temperature. After cells swelling, the cell pellet was resuspended in 3:1 methanol:acetic acid and fixed for 30-min on ice. Centrifuged and washed cell pellet two times with 3:1 methanol:acetic acid, and then supernatant was replaced by fresh 3:1 methanol:acetic acid. Cells were dropped onto slides and dried at room temperature, followed by staining with DAPI for 5-min. *405-nm* excitation images were perform by a Leica DMI6000 inverted microscope equipped with an HCX PL FL *100x* 1.4 NA oil-immersion.

### Statistical analysis

The quantitation results for the protein and mRNA levels and live cell imaging are presented as the mean ± standard error, except for the individual data sets. All statistical analyses were performed using Prism software (GraphPad). Any *p* value less than 0.05 is considered significant (***p < 0.001; **p < 0.01; *p < 0.05).

## SUPPLEMENTARY FIGURE


