# Supplementary material for: Sgo1 is a potential therapeutic target for hepatocellular carcinoma
Source: Oncotarget. 2015 Jan 6;6(4):2023–33. doi: 10.18632/oncotarget.2764 (PMC4385833; doi:10.18632/oncotarget.2764)
Supplement: Supplementary file 1 [file oncotarget-06-2023-s001.pdf]

SUPPLEMENTARY FIGURE

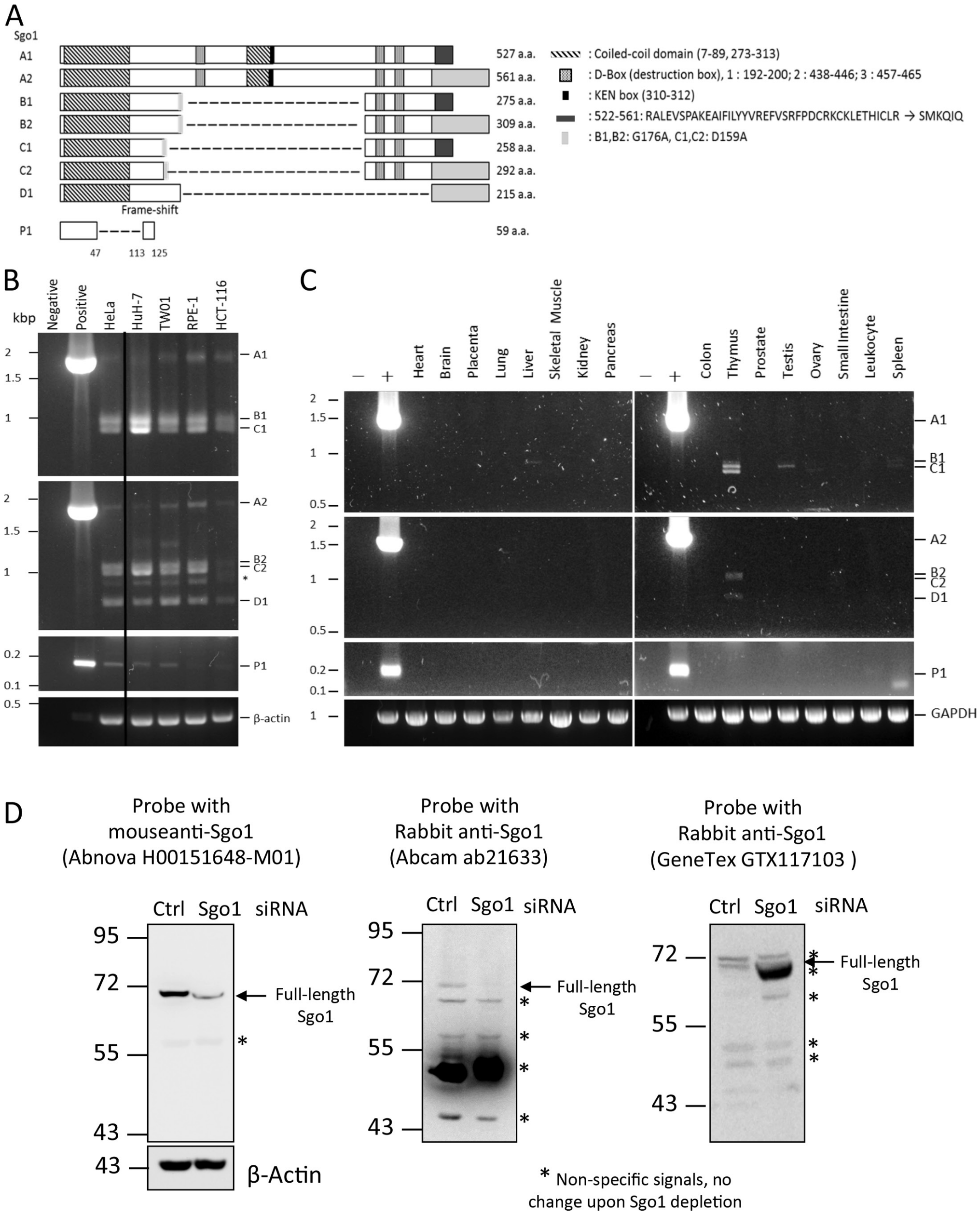

Supplementary Figure 1: Characterization for the expression of Sgo1 transcription splicing variants. (A) Scheme of Sgo1 splicing variants. Eight isoforms of Sgo1 were identified from database searches, including 2 full-length isoforms A1/A2 and 6 small variants B1/B2/C1/C2/D1/P1. **(B)** Three sets of specific primers were applied to detect various Sgo1 variants in HeLa, HuH-7, TW01, RPE-1, and HCT-116 cells by conventional RT-PCR. Most Sgo1 splicing variants were detected in these cell lines. **(C)** Multiple tissue cDNAs were amplified to detect different Sgo1 variants by RT-PCR. Sgo1 mRNA was only detected in thymus, testis, and spleen, with the majority being B1, B2, C1, and C2 variants. **(D)** Representative blots of HeLa cells depleted with control (Ctrl) and Sgo1. The same lysates were probed with three different antibodies, including Abnova (H00151648-M01), Abcam (ab21633) and GeneTex (GTX117103). Note that although various bands were detected in these blots, only the signal corresponding to 70 Kda was lost upon Sgo1 depletion. Thus, full-length Sgo1 is likely the only translated product of Sgo1 in HeLa cells. Similar results were observed in other cell lines (as shown in Figure 4A).

SUPPLEMENTARY VIDEO INFORMATION

Multi-dimensional time-lapse imaging was performed using a Leica DMI6000 inverted microscope equipped with an HCX PL FL 20x objective and an Andor Luca R EMCCD camera. Differential interference contrast images were acquired for follow-up image analysis. Representative videos illustrating cells treated with either control or Sgo1-specific siRNA are provided as follows. Time stamps displayed as 00:00 for hr:min.

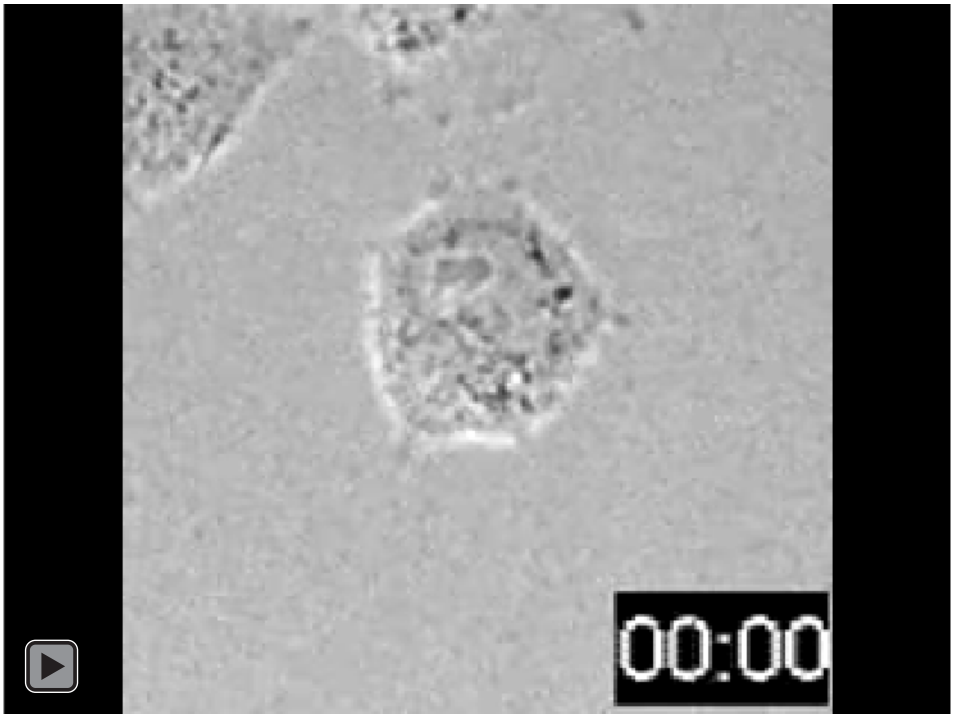

Supplementary Video S1: HeLa cells treated with Sgo1 siRNA.

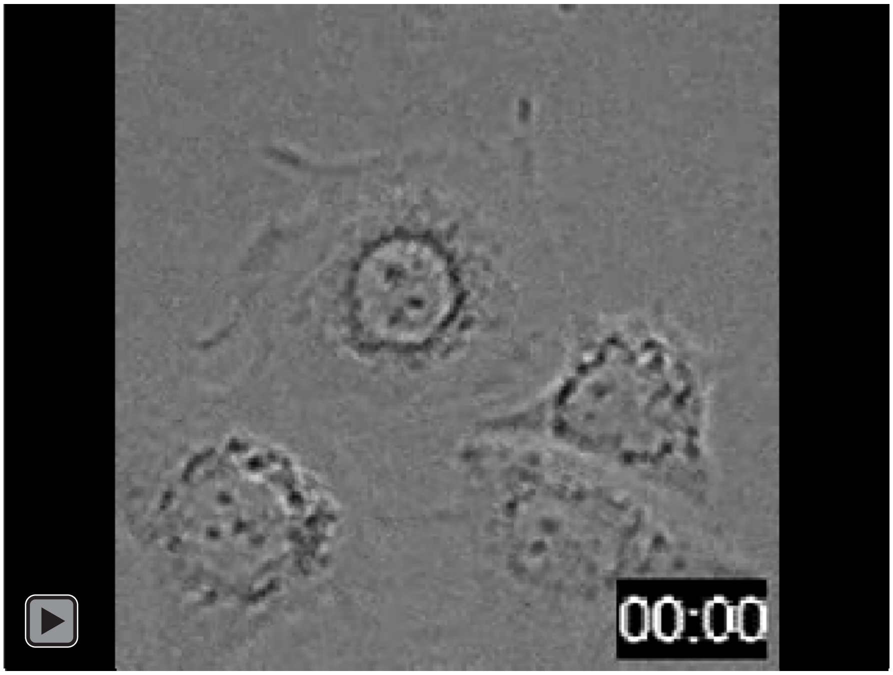

Supplementary Video S2: HuH-7 cells treated with control siRNA.

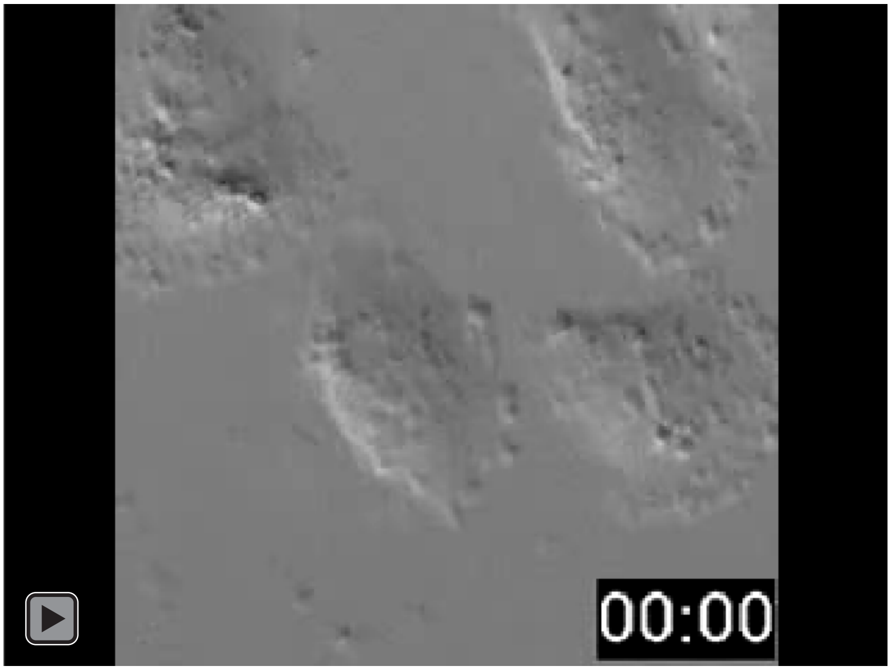

Supplementary Video S3: HuH-7 cells treated with Sgo1 siRNA.

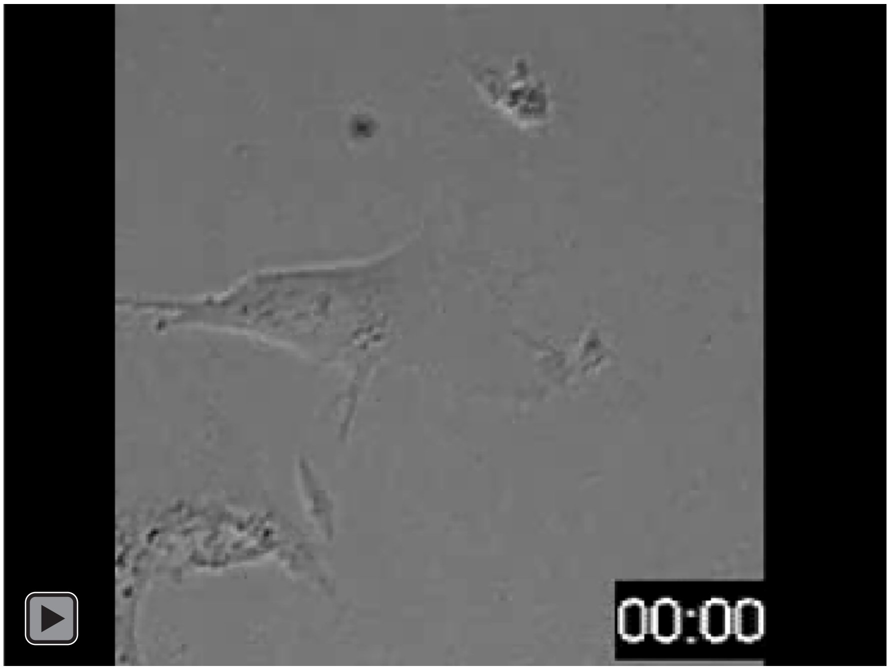

Supplementary Video S4: NeHepLxHT cells treated with control siRNA.

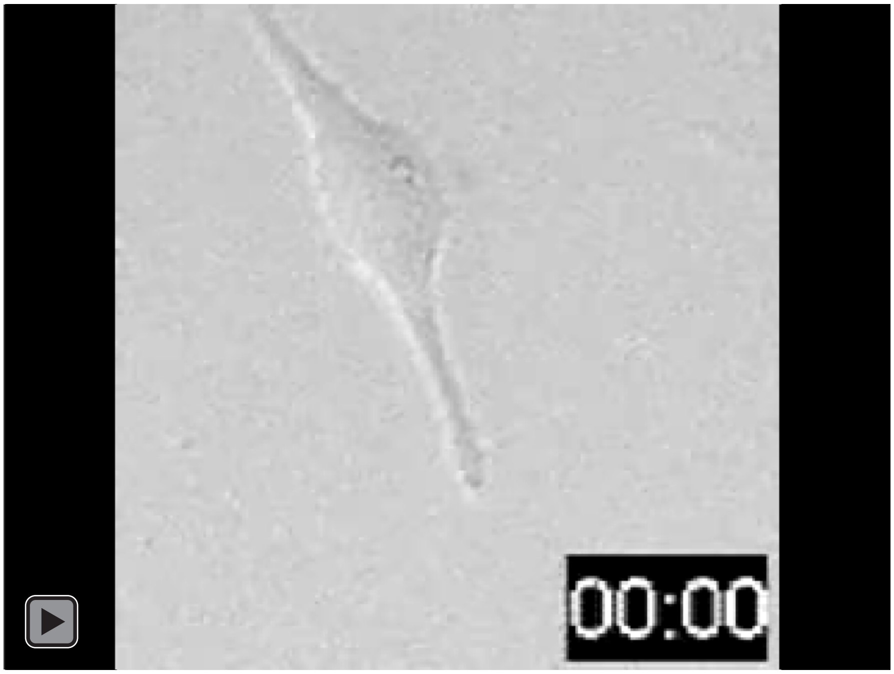

Supplementary Video S5: NeHepLxHT cells treated with Sgo1 siRNA.

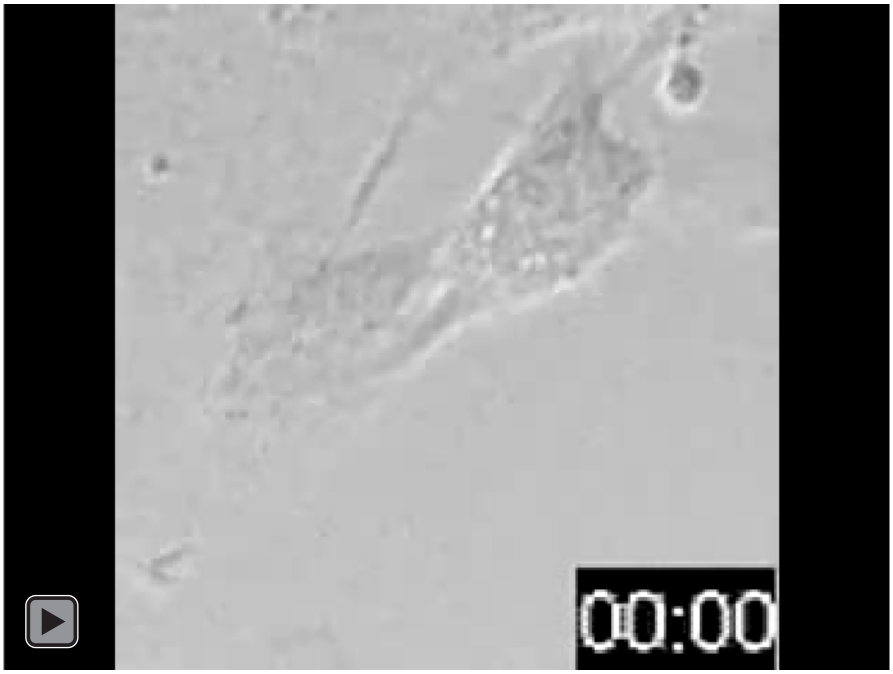

Supplementary Video S6: RPE-1 cells treated with Sgo1 siRNA.
